# Expression of *VEGFA*-mRNA in classical and *MSX2*-mRNA in non-classical monocytes in patients with spondyloarthritis is associated with peripheral arthritis

**DOI:** 10.1038/s41598-021-89037-2

**Published:** 2021-05-06

**Authors:** Małgorzata Stec, Michał Seweryn, Mariusz Korkosz, Zofia Guła, Rafał Szatanek, Kazimierz Węglarczyk, Magdalena Rutkowska-Zapała, Marzena Lenart, Marcin Czepiel, Jarosław Czyż, Jarosław Baran, Anna Gruca, Kamila Wojnar-Lasoń, Paweł Wołkow, Maciej Siedlar

**Affiliations:** 1 Department of Clinical Immunology, Institute of Pediatrics, Jagiellonian University Medical College, Wielicka 265 Str., 30-663 Kraków, Poland; 2 Center for Medical Genomics OMICRON, Jagiellonian University Medical College, Kopernika 7c Str., 31-034 Kraków, Poland; 3 Department of Rheumatology and Balneology, Jagiellonian University Medical College, Jakubowskiego 2 Str., Kraków, Poland; 4 Department of Cell Biology, Faculty of Biochemistry, Biophysics and Biotechnology, Jagiellonian University, Gronostajowa 7 Str., Kraków, Poland

**Keywords:** Immunology, Rheumatology

## Abstract

Spondyloarthritis (SpA) is characterized by chronic inflammation and structural damage involving spine and peripheral joints. Monocytes, as part of innate immune system, following migration into affected tissue, may play a role in the pathogenesis of SpA. Here, potential associations between osteogenesis-linked gene expression profile in particular monocyte subpopulations and clinical signs of SpA were investigated. The 20 patients with axial and 16 with peripheral SpA were enrolled in the study. Monocyte subpopulations (classical—CD14^++^CD16^−^, intermediate—CD14^++^CD16^+^ and non-classical—CD14^+^CD16^++^) were isolated from blood using flow cytometry and gene expression analysis was performed using real-time PCR method and TaqMan Array, Human Osteogenesis, Fast 96-well plates. Next, the characteristic clinical features shared by axial and peripheral SpA were analyzed in the context of the expression of selected genes in the three subpopulations of monocytes. We demonstrated that expression of *VEGFA* in classical and *MSX2* in non-classical monocytes were associated with the number of swollen and painful peripheral joints of SpA patients. We conclude that monocytes may contribute to the development of peripheral arthritis in SpA patients. This might be possible through subpopulation specific effects, linking number of inflamed joints with expression of *VEGFA* in classical monocytes and *MSX2* in non-classical monocytes.

## Introduction

Spondyloarthritis (SpA) represents a group of second most prevalent inflammatory rheumatic disorders (ca. 0.2–1.6% depending on geographic area)^1^ characterized by chronic inflammation and structural damage involving axial and peripheral skeleton. Recent research in SpA has been focused on the phenotypic presentations and pathophysiology of SpA subgroups (ie. non-radiographic axial spondyloarthritis, ankylosing spondylitis, psoriatic arthritis, reactive arthritis, arthritis in inflammatory bowel diseases and undifferentiated spondyloarthritis) exploring, whether SpA is a single disease with various clinical expression or rather a group of distinct clinical entities sharing common signs and symptoms^2^.

Genetic, immunopathologic, and clinical evidence indicate that despite common downstream pathways, mediated e.g. by macrophage-derived TNFα, inflammation in SpA is driven and maintained by different cellular and molecular mediators^3,4^. Moreover, it has been proposed that SpA is an autoinflammatory disease driven rather by innate immune cells, than a genuine autoimmune disease triggered by self-reactive T and/or B lymphocytes^5^. The phenotypic subclassification of SpA is usually based on extraarticular signs (psoriasis and inflammatory bowel disease), pathogenesis (reactive arthritis) or outcomes (ankylosing spondylitis)^2^. Nevertheless, all phenotypes share similar axial (sacroiliitis, spondylitis, back pain) or peripheral (arthritis, enthesitis, dactylitis) manifestations, and therefore SpA might be classified as one of two subforms with different pathophysiology, with predominant involvement of axial or peripheral skeleton^1^. In other words, there is a proposal to define SpA by its pathophysiology rather than by its phenotypic presentation, since the emerging data from immunopathology studies and clinical trials suggest that axial (axSpA) and peripheral (pSpA) spondyloarthritis might be driven by different mechanisms and respond differently to treatment, supporting the classification of SpA according to the presence of axial or peripheral disease^2^. However, considering SpA as a possible single entity the question remains whether shared clinical features of axSpA and pSpA could have common triggers, particularly during the early phase before chronic compensatory and therapy effects occur. Therefore, it is interesting to explore mechanisms leading to SpA manifestations shared by axSpA and pSpA. They are likely to be most variable and at the same time most informative at an early stage of the disease.

In such setting, pathophysiological role of monocyte subpopulations as a source of pro- and anti-inflammatory cytokines, bone remodeling proteins and other biologically active compounds is not fully elucidated. Moreover, there is some evidence that monocytes may be the source of novel bone forming cells (“monoosteophils”)^6^.

The aim of our study was to link characteristic clinical features seen in axSpA and pSpA with different expression of selected genes in three subpopulations of monocytes isolated from blood of SpA patients. We focused on the manifestations which could be expressed both in axSpA and pSpA—i.e. arthritis, enthesitis, dactylitis and inflammatory back pain. This might help to understand how monocytes and possibly derived from them macrophages and osteoclasts might drive particular pathological processes, which are then interpreted as characteristic clinical signs of both axSpA and pSpA.

## Results

### Demographic, clinical and laboratory data

Table 1 presents characteristics of patients. Briefly, median age (years, IQR) of axSpA patients was 33.5 (29.7–39.7) and pSpA patients was 35.5 (31–38.5). Median disease duration (years, IQR) was 7 (5–10.7) for axSpA and 3 (2–9.5) for pSpA patients. 95% of axSpA and 38% of pSpA patients were HLA-B27 positive.

**Table 1:** Demographic and clinical characteristics of patient groups.

	Axial SpA (n = 20)	Peripheral SpA (n = 16)	*p-*value
Age (years), median (IQR)	33.5 (29.7–39.7)	35.5 (31–38.5)	NS
Males, n (%)	14 (70)	8 (50)	< 0.001
HLA B27 positive n (%)	18 (95%)	5 (38%)	0.002
Disease duration (years), median (IQR)	7 (5–10.7)	3 (2–9.5)	NS
ESR mm/h, median (IQR)	24 (11–33)	25 (17–37)	NS
CRP mg/l, median (IQR)	7.02 (1.4–11.98)	8.02 (3.98–13.7)	NS
BASDAI median (IQR)	2.5 (1.1- 4.6)	4.9 (2.9–6.4)	NS
ASDAS (CRP) median (IQR)	2 (1.5–3.0)	3.1 (2.1–3.5)	NS
IBP (total back pain), n (%)	16 (80)	6 (40)	0.03
Number of swollen joints (out of 66), median (IQR)	0	2 (1–4)	< 0.001
Number of painful joints (out of 68), median (IQR)	0	2 (0–4)	< 0.001
DAS28 (ESR) median (IQR)	NA	4.0 (3.0–4.4)	NA
Enthesitis n (%)	7 (35)	10 (71)	NS
Dactylitis n (%)	2 (10)	14 (93)	< 0.001
**Diagnosis, n (%)**			
nr axSpA	5 (25)	0	
AS	15 (75)	0	
PsA	0	8 (50)	
per SpA	0	8 (50)	

*BASDAI* Bath Ankylosing Spondylitis Disease Activity Index; *ASDAS* Ankylosing Spondylitis Disease Activity Score; *DAS28* Disease Activity Score (ESR) 28; *IBP* inflammatory back pain; *CRP* C-reactive protein; *ESR* erythrocyte sedimentation rate; *ax* axial; *nr* non radiographic; *per* peripheral; *SpA* spondyloarthritis; *AS* ankylosing spondylitis; *PsA* psoriatic arthritis.

Fifteen axSpA patients fulfilled mNY criteria for ankylosing spondylitis.

### Selected gene and probe panels are differently expressed among three monocyte subpopulations

**Figure 1:**
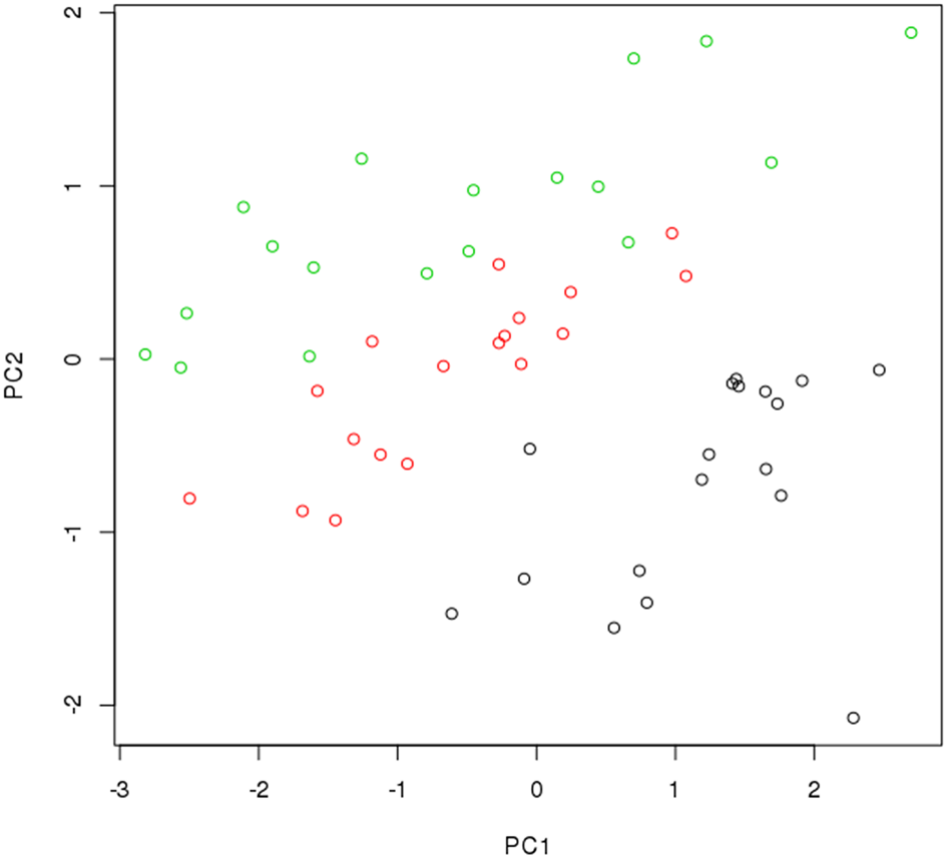
The results of PC analysis of microarray expression data in classical (black dots), non-classical (green dots) and intermediate (red dots) monocytes. Only probes in genes whose expression was initially measured (and expressed well on the array) were selected for this analysis. PC1 and PC2 are the first two principal components estimated based on the expression data with the aid of the prcomp function in R^49^.

To explore the expression of mRNAs tested in our panel across monocyte subpopulations in SpA we utilized microarray expression data generated in a previous study by Metcalf et al.^7^ (18 individuals, 3 subsets of monocytes per sample). We acknowledged 94 genes (154 probes) which constituted our mRNA SpA panel and then using a Principal Component Analysis we identified 3 clusters of samples—each corresponding to different subpopulation of monocytes as shown in Fig. 1.

### Associations between mRNAs and clinical signs

We analyzed whether mRNAs identified in different monocyte subpopulations were correlated with clinical signs of axSpA and pSpA. The following were selected: (1) inflammatory back pain (total back pain, BASDAI question 2, range 1–10), and (2) number of swollen joints (out of 66 total joint count and out of 28 joint count from DAS28 score), (3) number of painful joints (out of 68 total joint count and out of 28 joint count from DAS28 score), (4) presence of enthesitis and (5) presence of dactylitis.

### Expression of *VEGFA* in classical monocytes is associated with the number of swollen and painful joints

We found Vascular Endothelial Growth Factor A *(VEGFA)* mRNA in classical monocytes to be positively associated with measurement of joint involvement, i.e. number of swollen joints from total joint count and number of swollen and painful joints from DAS28 score, with False Discovery Rate (FDR) < 0.05 for each feature. The most robust association, with FDR < 0.001 was observed for the number of swollen joints from DAS28 score (Table 2).

**Table 2:** Results of the association analysis between clinical signs (swollen/painful) and gene expression in subsets of monocytes.

Gene	Monocyte subset	Number of joints	FDR	logFC
VEGFA	Classical	Swollen (total)	0.043	− 0.448
VEGFA	Classical	Painful (total)	0.191	− 0.412
VEGFA	Classical	Swollen (DAS28)	< 0.001	− 0.674
VEGFA	Classical	Painful (DAS28)	0.008	− 0.704
MSX2	Non-classical	Swollen (total)	0.002	− 0.956
MSX2	Non-classical	Painful (total)	0.083	− 0.906
MSX2	Non-classical	Swollen (DAS28)	< 0.001	− 1.072
MSX2	Non-classical	Painful (DAS28)	0.008	− 1.131

The false discovery rate (FDR) was estimated for each clinical variable separately due to small sample size.

*FDR* estimated false discovery rate based on all tests performed, *logFC* logarithm of the fold change of the expression levels between experimental conditions.

### Expression of *MSX2* in non-classical monocytes is also associated with peripheral arthritis

We identified the Muscle Segment Homeobox 2 (*MSX2)* mRNA expression in non-classical monocytes as positively associated with measurements of peripheral arthritis, i.e. number of swollen joints from total joint count and number of swollen and painful joints from DAS28 score, under the FDR < 0.08 for each feature (Table 2).

There were no significant associations between the expression of selected genes in the intermediate subset of monocytes with the number of swollen and painful joints. There were either no associations between the expression of *VEGFA* or *MSX2* mRNAs in subsequent monocytes subpopulations with other SpA clinical features, e.g. inflammatory back pain, enthesitis or dactylitis.

## Discussion

Here, we provide evidence that monocytes, cells of innate immune system, may contribute to the development of peripheral arthritis in SpA and therefore might be at play when considering chronic inflammation, one of the most significant features of this disease.

There is a substantial evidence concerning the role of the innate immune system in the pathogenesis of a variety of SpA features, including chronic inflammation, repair and new bone formation^8,9^. It seems that peripheral blood monocytes in SpA are functionally primed and/or functionally reprogrammed by not yet well identified factor(s) and exhibit molecular or cellular features characteristic for SpA^10–12^. Monocytes are classified into three subpopulations—classical (CD14^++^CD16^−^), intermediate (CD14^++^CD16^+^) and non-classical (CD14^+^CD16^++^); the latter two subpopulations being referred to as “proinflammatory”^13^. The “proinflammatory” subsets form app. 5–15% of circulating monocytes, express and secrete upon stimulation by various factors large amounts of pro-inflammatory cytokines, e.g. TNFα, interleukins (IL)—IL-12 and IL-1, but insignificant amount of anti-inflammatory IL-10^14^. Contrariwise, classical monocytes produce relatively small amounts of TNFα, but they are a robust source of IL-10^15^. Under physiological conditions most of classical monocytes (ca. 80–90%) leave the bloodstream after a circulating lifespan of 1 day, whereas remaining fraction further mature into intermediate cells and finally convert (in app. 12 days) to non-classical monocytes before leaving the circulation^16^. Then extravasated monocytes supplement the population of resting tissue macrophages, but they may differ functionally. These cells may influence inflammatory processes by factors released inside blood vessels or locally, upon their migration^17^.

Currently, there are only a few studies (based on the analysis of single blood samples) that investigated differences in transcriptome/proteome profile of monocyte subpopulations isolated from healthy individuals [e.g.^18–25^]. These studies indicate, on molecular basis, the significant differences in genetic profiles between these three monocyte subpopulations, confirming and extending most of the former phenotypic and functional observations. However, there is no data so far comparing gene expression in monocyte subsets in SpA patients exhibiting axial or peripheral signs.

Moreover, we have previously shown, that there were no differences in numbers of classical, intermediate and non-classical monocytes between axSpA and pSpA patients. There were also no differences concerning numbers of intermediate and non-classical monocytes between SpA patients and controls. The only statistically significant difference was seen between SpA patients and controls regarding classical monocytes^26^.

Vascular endothelial growth factor-A (VEGFA) is one of the most important growth factors engaged in vascular development and angiogenesis. Since bone is a highly vascularized organ and angiogenesis plays an important role in osteogenesis, it has been established that VEGFA also influences skeletal development and postnatal bone repair^27^. Process of bone remodeling is based on a balance between bone formation and resorption^28^. Disturbance of this balance may strongly depend on both osteoclastic and osteoblastic activity. Liu et al.^29^ suggested, that VEGFA and TNFα might directly participate in the differentiation of fibroblasts into osteoblasts, and anti-VEGFA has been suggested as a possible new therapy preventing osteogenesis in SpA patients^28^.

We have shown that *VEGFA*-mRNA expression in classical monocytes positively correlated with the number of swollen and painful joints. Our results also confirmed higher VEGFA level in SpA patients’ sera when compared to healthy blood donors (Supplementary Fig. 1) supporting the previous findings by Lin et al.^30^. This may identify the classical monocyte subpopulation as the main source of VEGFA, which, upon migration to the synovium of peripheral joints, may promote local inflammation in SpA patients. It was already shown that VEGFA serum and synovial fluid levels are elevated in patients with ankylosing spondylitis expressing features of peripheral arthritis^31,32^. Moreover, VEGFA may be secreted by various cell types, including macrophages, which are present in the synovial membrane and entheses in patients with SpA, but no association with particular monocyte subpopulation (as a source of particular tissue macrophages) has been specified^32^.

Whereas the role of VEGF in the etiopathogenesis of SpA may be pragmatically interpreted on the ground of former scientific observations and is clinically proven, our second finding referring to the MSX2 is not so easy interpretable. MSX2 is a transcription factor with a homeobox domain, presumably involved in bone development and ectopic calcifications, although its role in these processes is still controversial^33–42^. Furuichi et al. examined a total of 45 single nucleotide polymorphisms (SNPs) in 15 genes by sequential screening and reported promising evidence for the association between *MSX2* polymorphisms and SpA in Japanese population^33^. Moreover, in the basic studies involving animal models, the *MSX2* knockout mice displayed remarkable decrease in mineralization of the axial skeleton, reduced proliferation of osteoprogenitors defecting skull ossification and abnormal calvarial development^34^, whereas transgenic mice overexpressing *MSX2* showed enhanced proliferation of calvarial cells^35,36^. A loss-of-function mutation of *MSX2* in humans, which reduces DNA binding activity, causes defect in skull ossification^37^. These observations contrast with a gain-of-function mutation of *MSX2,* which results in an autosomal dominant disorder, Boston-type craniosynostosis^38,39^. These findings demonstrate that *MSX2* expression is critical for human skull development and suggest its positive ossific role in bone development. However, MSX2 protein suppresses the expression of bone marker genes, including RUNX2 (a master regulator of osteoblast differentiation) and osteocalcin, and negatively regulates bone development and ectopic calcification^40–42^. The roles of MSX2 may vary depending on cell type and/or cell differentiation stage.

We have demonstrated that *MSX2*-mRNA overexpression in non-classical monocytes is positively associated with the number of swollen and painful joints, and therefore question the canonical role of this protein expressed preferentially in non-classical subset of monocytes. It was shown that TNFα may induce *MSX2* expression and MSX2 mediates the inhibitory action of TNFα in osteoblast differentiation^43,44^. It may not be excluded that MSX2 expression in non-classical monocytes is secondary to their proinflammatory functions related to TNFα auto- or paracrine action. Moreover, upon migration into soft tissue, non-classical monocytes may contribute to local inhibition of the BMP2-regulated osteoblast differentiation within inflamed peripheral joints^44^ and therefore being possibly involved in new bone formation and joint remodeling. These concepts must be verified applying the appropriate mouse model (e.g. SKG mice) of SpA associated peripheral arthritis, which is currently underway.

Our study has some limitations. Obviously, the number of patients is small, but it is our belief that this pilot observation is interesting although requires further investigation. Also, this is a cross-sectional study and we do not know whether the discovered findings are durable enough to be attributed to chronic pain and synovitis characteristic for pSpA. Finally, we were exploring the peripheral blood monocytes only, having no matching data considering the local environment of synovial tissue.

## Conclusions

Our data suggest that monocytes may contribute to the development of peripheral arthritis in SpA patients. This might be due to the subpopulation specific effects, linking the number of swollen and painful joints with the expression of VEGFA in classical monocytes and MSX2 in non-classical monocytes. We argue for the first time that overexpression of both proteins in classical and non-classical subsets of monocytes may be linked with the inflammatory process within peripheral joints in SpA patients.

## Methods

### Patients

Thirty-six patients with SpA (20 axSpA and 16 pSpA) according to the Assessment of SpondyloArthritis International Society classification criteria^45,46^ were enrolled in the study. Patients were under 45 years, naive to synthetic, synthetic-targeted or biologic Disease Modifying Anti-Rheumatic Drugs (DMARDs) and without administration of systemic glucocorticosteroids. Patients provided a signed informed consent and the study protocol was approved by the local bioethics committee and all methods were performed in accordance with the relevant guidelines and regulations.

### Isolation of monocytes and their subsets

Monocyte subpopulations were isolated from peripheral blood mononuclear cells (PBMC) obtained from SpA patients. PBMC were isolated from EDTA-treated whole peripheral blood by the standard Pancoll human (PAN-Biotech, Aidenbach, Germany) density gradient centrifugation. PBMC were washed in PBS (Sigma-Aldrich, Saint Louis, USA) and then monocyte subsets (classical—CD14^++^CD16^−^, intermediate—CD14^++^CD16^+^ and non-classical—CD14^+^CD16^++^) were isolated using flow cytometry cell sorting. The following monoclonal antibodies (mAbs) were used to stain monocytes: anti-CD14-FITC (clone MφP9, BD Biosciences, San Jose, CA, USA), anti-CD16-PE (clone 3G8, BD Biosciences) and anti-HLA-DR-PerCP (clone L243, BD Biosciences), in 1:25 dilution v/v stained and gated as previously described by us and others^47,48^. The stained monocytes were then incubated for 30 min at 4 °C after which they were sorted using the FACSAria II cell sorter (BD Biosciences). Sorter was equipped with 488 nm laser for excitation of FITC, PE and PerCP. The following band-pass filters were used for the measurement of fluorescence: 530/30 for FITC, 582/42 for PE and 695/40 for PerCP. After isolation, the cells were washed in PBS, centrifuged for 10 min at 350×*g* and kept frozen at − 80 °C until RNA isolation.

### Gene expression analysis using real-time PCR

Gene expression analysis was performed using real-time PCR method and TaqMan Array, Human Osteogenesis, Fast 96-well plates, # 4418741 (coverage of 92 osteogenesis associated genes and 4 endogenous control genes, Supplementary Table 1) (Thermo Fisher Scientific, Waltham, MA, USA). RNAs were isolated using miRVana microRNA isolation kit (Thermo Fisher Scientific); isolated RNA were transcribed into cDNA using Superscript IV VILO mastermix (Thermo Fisher Scientific). Next, cDNA was used for the assessment of gene expression profile using the TaqMan Array Human Osteogenesis plate and QuantStudio 3 Real-Time PCR instrument (Thermo Fisher Scientific) according to manufacturer’s protocol.

### Statistical methodology

All statistical analyses, as well as data pre-processing, normalization and visualization was done in R (version 3.5.2)^49^. The expression was calculated as the Ct values and filtered according to manufacturer’s instructions (only values with high confidence scores were used for further analyses). Subsequently, data was divided into three panels—each corresponding to a different subpopulation of monocytes: (1) classical, (2) intermediate, and (3) non-classical. The expression values were further normalized using the quantile normalization method as implemented in the package ‘preprocessCore’ (version 1.44.0). For the target statistical analysis, only genes with at least 5 observations in a given panel were used. In return, 88 genes were selected for further analysis in 37 samples of non-classical monocytes, and 38 samples of classical as well as intermediate monocytes. The association between axial or peripheral signs and gene expression in subpopulations of monocytes was tested via a simple linear model (with intercept and one predictor only) with empirical Bayes correction as implemented in the package ‘limma’ (version 3.38.3).

### Ethics approval and consent to participate

The study protocol was approved by the local bioethics committee (KBET/252/B/2012, Bioethics Committee of the Jagiellonian University, Podwale 3 Str., 31-118 Krakow, Poland). All included patients gave their informed written consent.

## Supplementary Information

Supplementary Figure S1.

Supplementary Table S1.
