# Supplementary material for: Expression of VEGFA-mRNA in classical and MSX2-mRNA in non-classical monocytes in patients with spondyloarthritis is associated with peripheral arthritis
Source: Sci Rep. 2021 May 6;11:9693. doi: 10.1038/s41598-021-89037-2 (PMC8102490; doi:10.1038/s41598-021-89037-2)
Supplement: Supplementary file 1 — Supplementary Figure S1. [file 41598_2021_89037_MOESM1_ESM.docx]

Supplementary Fig. 1. **Serum VEGFA concentration in SpA patients and controls.** Serum for cytokines assessment were obtained from SpA patients (23) and controls (13) and stored at -20^0^C. By means of enzyme-linked immunosorbent assays (ELISA) levels of VEGFA (Thermo Fisher Scientific) were assessed. Samples were run in duplicates, according to the manufacturer and results were obtained using the ELISA reader (BioTek Instruments, Winooski, VT, USA). Detection level for VEGFA was 7,9pg/ml. Compared with controls, patients with SpA had significantly higher concentration of VEGFA, p=0,03 (t Test).
