# Supplementary material for: Expression of VEGFA-mRNA in classical and MSX2-mRNA in non-classical monocytes in patients with spondyloarthritis is associated with peripheral arthritis
Source: Sci Rep. 2021 May 6;11:9693. doi: 10.1038/s41598-021-89037-2 (PMC8102490; doi:10.1038/s41598-021-89037-2)
Supplement: Supplementary file 2 — Supplementary Table S1. [file 41598_2021_89037_MOESM2_ESM.doc]

Supplementary Table 1. The panel of genes studied. A- assay ID, B-gene symbol

A

B
